# Identification of potential non-invasive biomarkers in diastrophic dysplasia

**DOI:** 10.1016/j.bone.2023.116838

**Published:** 2023-07-16

**Authors:** Chiara Paganini, Ricki S. Carroll, Chiara Gramegna Tota, Andrea J. Schelhaas, Alessandra Leone, Angela L. Duker, David A. O’Connell, Ryan F. Coghlan, Brian Johnstone, Carlos R. Ferreira, Sabrina Peressini, Riccardo Albertini, Antonella Forlino, Luisa Bonafé, Ana Belinda Campos-Xavier, Andrea Superti-Furga, Andreas Zankl, Antonio Rossi, Michael B. Bober

**Affiliations:** a Department of Molecular Medicine, Unit of Biochemistry, University of Pavia, Pavia, Italy; b Nemours Children’s Hospital, Wilmington, DE, USA; c Thomas Jefferson University, Philadelphia, PA, USA; d University School for Advanced Studies Pavia, IUSS Pavia, Pavia, Italy; e Shriners Hospitals for Children, Portland, OR, USA; f Oregon Health and Science University, Portland, OR, USA; g National Human Genome Research Institute, NIH, Bethesda, MD, USA; h Laboratory of Clinical Chemistry, Fondazione IRCCS Policlinico San Matteo, Pavia, Italy; i Division of Genetic Medicine, Centre Hospitalier Universitaire Vaudois, University of Lausanne, Switzerland; j University of Sydney, The Children’s Hospital at Westmead and Garvan Institute for Medical Research, Sydney, Australia

**Keywords:** Biomarker, Collagen X, Diastrophic dysplasia, Glycosaminoglycan, Sulfation, Urine

## Abstract

Diastrophic dysplasia (DTD) is a recessive chondrodysplasia caused by pathogenic variants in the *SLC26A2* gene encoding for a cell membrane sulfate/chloride antiporter crucial for sulfate uptake and glycosaminoglycan (GAG) sulfation. Research on a DTD animal model has suggested possible pharmacological treatment approaches. In view of future clinical trials, the identification of non-invasive biomarkers is crucial to assess the efficacy of treatments. Urinary GAG composition has been analyzed in several metabolic disorders including mucopolysaccharidoses. Moreover, the N-terminal fragment of collagen X, known as collagen X marker (CXM), is considered a real-time marker of endochondral ossification and growth velocity and was studied in individuals with achondroplasia and osteogenesis imperfecta. In this work, urinary GAG sulfation and blood CXM levels were investigated as potential biomarkers for individuals affected by DTD. Chondroitin sulfate disaccharide analysis was performed on GAGs isolated from urine by HPLC after GAG digestion with chondroitinase ABC and ACII, while CXM was assessed in dried blood spots. Results from DTD patients were compared with an age-matched control population. Undersulfation of urinary GAGs was observed in DTD patients with some relationship to the clinical severity and underlying *SLC26A2* variants. Lower than normal CXM levels were observed in most patients, even if the marker did not show a clear pattern in our small patient cohort because CXM values are highly dependent on age, gender and growth velocity. In summary, both non-invasive biomarkers are promising assays targeting various aspects of the disorder including overall metabolism of sulfated GAGs and endochondral ossification.

## 1. Introduction

Diastrophic dysplasia (DTD, MIM #222600) is a rare autosomal recessive disorder of cartilage and bone development. Incidence is unknown but predicted to be 1:100,000 placing DTD among rare genetic disorders [1]. The incidence of DTD in Finland is approximately 1:33,000 due to the presence of a founder variant [2]. DTD results from pathogenic homozygous or compound heterozygous variants in the *SLC26A2* gene, also known as the diastrophic dysplasia sulfate transporter (*DTDST*) gene. *SLC26A2* encodes for a transmembrane sulfate transporter [3]. Variants in *SLC26A2* give rise to a spectrum of skeletal dysplasias that results from the reduction of sulfate transport and subsequent undersulfation of cartilage proteoglycans (PGs) essential to endochondral bone and articular cartilage formation. Phenotypic severity correlates with the level of residual sulfate transport and degree of PG undersulfation, which ranges from *in utero* lethal achondrogenesis type 1B (ACG1B, MIM #600972), to atelosteogenesis type 2 (AO2, MIM #256050), to DTD and to recessive multiple epiphyseal dysplasia (rMED; MIM#226900) [4,5].

DTD is a clinically and radiographically well-characterized skeletal dysplasia that, in most cases, does not require genetic testing to diagnose. Skeletal manifestations of DTD comprise spondyloepimetaphyseal dysplasia with mesomelia, cervical kyphosis, progressive kyphoscoliosis, lower extremity deformities including bilateral hip dysplasia, patellar dislocation, foot deformities, and ulnar deviation of the second digit with abduction of the thumb, also known as hitchhiker thumb [6,7]. Clinically, cleft palate, tracheomalacia and cystic swelling of the external ear may be observed [6]. Progressive joint contractures and osteoarthritis in young adulthood result in mobility impairment [8–10]. Intelligence is normal [6].

There is no pharmacological treatment for DTD; care is symptomatic and with a primary focus on environmental modifications to support activities of daily living, by physiotherapy, and surgical intervention [10–12]. Recent studies in the dtd mouse, an animal model of human DTD, have shown promising results including improvements in skeletal phenotype and anthropometric measurements after treatment with *N*-acetylcysteine (NAC), which acts as an intracellular source of sulfate in the prenatal and postnatal periods [13,14]. To improve the pharmacokinetics of NAC for a potential clinical trial, specific formulation including polyethylene glycol-poly-lactide-co-glycolide block copolymer (PLGA-PEG) nanoparticles are under investigation [15]. These studies represent the potential for repurposing a drug that is approved by various health authorities including the FDA and EMA to treat other medical conditions. Another study in *slc26a2* knock-out mice resembling ACG1B and AO2 phenotypes demonstrated that suppression of over-activated FGFR3 signaling by inhibition of FGFR3 or its downstream effectors improved cartilage growth *in vitro* as well as the skeletal phenotype of affected newborn mice born from treated pregnant females [16]. These findings suggest that there is potential for the pharmacological treatment of affected individuals. At present, no DTD-related biomarkers are available. For this reason, non-invasive biomarkers that, together with the clinical data, can provide information on treatment efficacy are needed.

Urinary glycosaminoglycans (GAGs) result from the turnover of PG in the extracellular matrix (ECM). The analysis of urinary GAGs was used as a biomarker to evaluate the efficacy of enzyme replacement therapy in individuals with mucopolysaccharidoses (MPS), a group of lysosomal storage disorders [17,18]. Furthermore, urinary GAG analysis can be used to detect and monitor the clear cell renal cell carcinoma and bladder carcinoma [19,20]. In the dtd mouse, the undersulfation of urinary GAGs was demonstrated when compared with wild-types in newborns and in 1- and 2-month-old mice [13,21].

Recently, it has been demonstrated that collagen X marker (CXM), a byproduct of collagen X degradation, serves as a serum biomarker which strongly correlates with the growth velocity in typically-growing children [22]. Based on this observation, serum CXM levels were analyzed in a cohort of individuals with achondroplasia, and were decreased when compared with age- and sex-matched controls [23]. Altered serum levels were also detected in individuals with osteogenesis imperfecta [24].

Given individuals with DTD have impaired growth velocity, joint and growth plate abnormalities and defects in PG sulfation, in this work, urinary GAG sulfation and CXM blood levels were measured for the first time as potential biomarkers in affected children.

## 2. Materials and methods

### 2.1. Study population and sample collection

Individuals with either a clinical and radiograph diagnosis (patient ID from 18 to 21) and a genetically confirmed diagnosis of DTD (patient ID from 1 to 17) were recruited for the study. Written parental consent was obtained for all children. Clinical data was collected for all study participants; urine was collected from 16 individuals ages 0.2 to 12 years, while dried blood spot (DBS) samples were collected from 15 individuals between 0.5 and 14 years old. Urine samples were not available for patient ID from 17 to 21, while DBS were not available for patient ID 1, 6, 9, 10, 13 and 14. All samples were obtained prospectively under protocols approved by the Institutional Review Board at Nemours Children’s Hospital, Wilmington, Delaware and Thomas Jefferson University, Philadelphia, Pennsylvania, USA, Centre Hospitalier Universitaire Vaudois, University of Lausanne, Switzerland, and The Children’s Hospital at Westmead and Garvan Institute for Medical Research, Sydney, Australia. Samples were shipped in dry ice and stored at −80°C.

### 2.2. Control population

Residual urine samples collected for clinical purposes from unaffected children ages 0.04 to 11 years at Policlinico San Matteo, Pavia, Italy were analyzed anonymously as controls. All children had written parental permission obtained to use urine samples for research. CXM reference data were obtained from previously published normative data in unaffected individuals [22].

### 2.3. Urine sulfation assay

Sulfation analysis of urinary GAGs was performed as previously reported [25,26]. Urine from affected individuals and age-matched controls were clarified by centrifugation at 13000 ×*g* for 15 min and GAGs were recovered by 0.2 % cetylpyridinium chloride precipitation at 4 °C overnight. Then samples were centrifuged at 13000 ×*g* for 15 min. The pellets were washed three times with 10 % potassium acetate in 96 % ethanol and three times with 96 % ethanol. GAGs were then resuspended in 0.1 M ammonium acetate, pH 7.35, and digested with 30 mU chondroitinase ABC (AMSBIO) and 30 mU chondroitinase ACII (Sigma-Aldrich) at 37 °C overnight to release chondroitin sulfate disaccharides. Then samples were lyophilized and disaccharides derivatized with 2-aminoacridone as previously described [13]. Samples were analyzed by an HPLC binary pump system (Binary HPLC Pump 1525 μ, Waters) coupled to a fluorescent detector (Multi λ Fluorescence detector 2475, Waters) set at λ_ex_ 425 nm and λ_em_ 525 nm. Chromatography was performed with a LiChroCART^®^ 250–4 Superspher^®^ 100 RP-18 endcapped column (Merck) and a LiChroCART^®^ 4–4 LiChrospher^®^ 100 RP-18 endcapped (Merck) as precolumn. Elution was carried out at room temperature at a flow rate of 0.7 ml/min with a linear gradient of 0.1 M ammonium acetate (VWR), pH 7.00, and methanol (VWR) as previously reported [14].

### 2.4. CXM assay

The CXM ELISA assay protocol has been well described [22,27]. The protocol used for DTD and control samples is identical to the CXM assay protocol previously outlined for individuals with achondroplasia [23]. To summarize, DBS samples were punched and eluded a 1:200 dilution in CXM sample diluent. Two 100 μl of the diluted samples, calibrators and associated controls were run on CXM ELISA plates. Concentration was generated using a 4 parameter logistic (4PL) nonlinear regression model of calibration curve with BioTek Gen5 software (R^2^ > 0.95 was determined to be acceptable).

### 2.5. Statistical analysis

For urinary GAG sulfation analysis data are reported as mean ± standard deviation (SD). To evaluate differences among groups, Student’s *t*-test was applied and two-tailed *p*-value < 0.05 was considered significant. Individual z-scores for urinary GAG sulfation in DTD individuals were calculated by measuring residuals between DTD cohort data and the control population considering mean and SD of each age group. Linear regression and sulfation analyses were performed using SigmaPlot 14 software. The difference between correlation coefficients was analyzed by Fisher’s r-to-Z transformation. Reference data for CXM levels in healthy growing individuals were acquired from previously published norms [22]. A nonparametric Nadaraya-Watson kernel regression with a smoothing bandwidth of ±2.5 years was computed on reference data to minimize sources of type I error while comparing with DTD levels [23]. Individual z-scores for CXM levels in DTD individuals were calculated by measuring residuals between DTD cohort data and the nonparametric regression estimate at each corresponding age. Age-dependent standard deviations for reference data were computed using the same ±2.5 years bandwidth for each of the 15 DTD individuals. Kernel regression was computed using RStudio (Version 1.4.1103, ©2009–2021 RStudio, PBC).

## 3. Results

### 3.1. Participant characteristics

Available *SLC26A2* genetic variants and the associated clinical features of each study participant are summarized in Table 1. Urine for sulfation study were available from 8 males and 8 females ranging in age from approximately 0.2 to 12 years old. Eight males and 7 females between 0.5 and 14 years of age participated in CXM analysis. Ten individuals were involved in both the urine sulfation and CXM analyses. Given the spectrum and variability of phenotypes, we stratified the participants into mild, moderate, and severe groupings (Table 1). Eight participants were placed into the severe group for the following criteria: severe spinal deformities requiring surgery and/or cervical kyphosis measuring >60° (which may not have required surgical correction) with respiratory failure requiring tracheostomy with mechanical ventilation. Eight participants were placed into the mild group for the following criteria: no involvement of the cervical, thoracic or lumbar spine which required any surgical intervention, and the stature of the participant was above the mean on Horton DTD linear growth grid [28]. The remaining 5 participants were placed into the moderate group. These participants were neither mild nor severe and in general had spinal involvement of medical significance.

### 3.2. Urinary GAG sulfation analysis

**Fig. 1.**
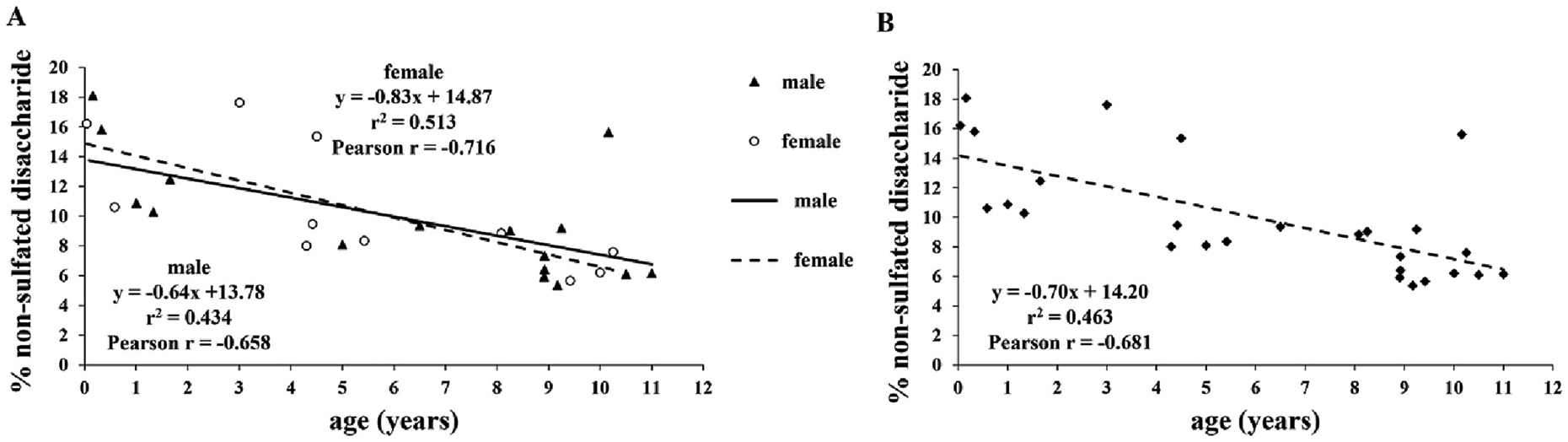
Correlation between the relative amount of non-sulfated disaccharide of chondroitin sulfate extracted from urine and age in controls. (A) Percentage of non-sulfated disaccharide for males and females plotted separately *vs*. age. Best fit linear regression lines were overlaid. (B) Male and female data were combined; best fit linear regression line was overlaid.

Urinary GAG sulfation was measured based on the relative amount of the non-sulfated disaccharide of chondroitin sulfate (ΔDi-0S) to the total chondroitin sulfate disaccharides (ΔDi-0S + ΔDi-4S + ΔDi-6S) following enzymatic depolymerization by chondroitinase ABC and ACII. Since reference data for urinary GAG sulfation were not available in children, 27 unaffected controls (16 males and 11 females) ranging from 0.04 to 11 years old were recruited in the study. A moderate correlation between urinary GAG sulfation and age was observed in the control population as demonstrated by the corresponding Pearson correlation coefficient (−0.658 and −0.716 for males and females, respectively, Fig. 1A). The Fisher Z transformation test found that the difference in correlation coefficients was not significant (z = 0.245, *P* = 0.806); therefore, results from both genders were combined (Fig. 1B).

**Fig. 2.**
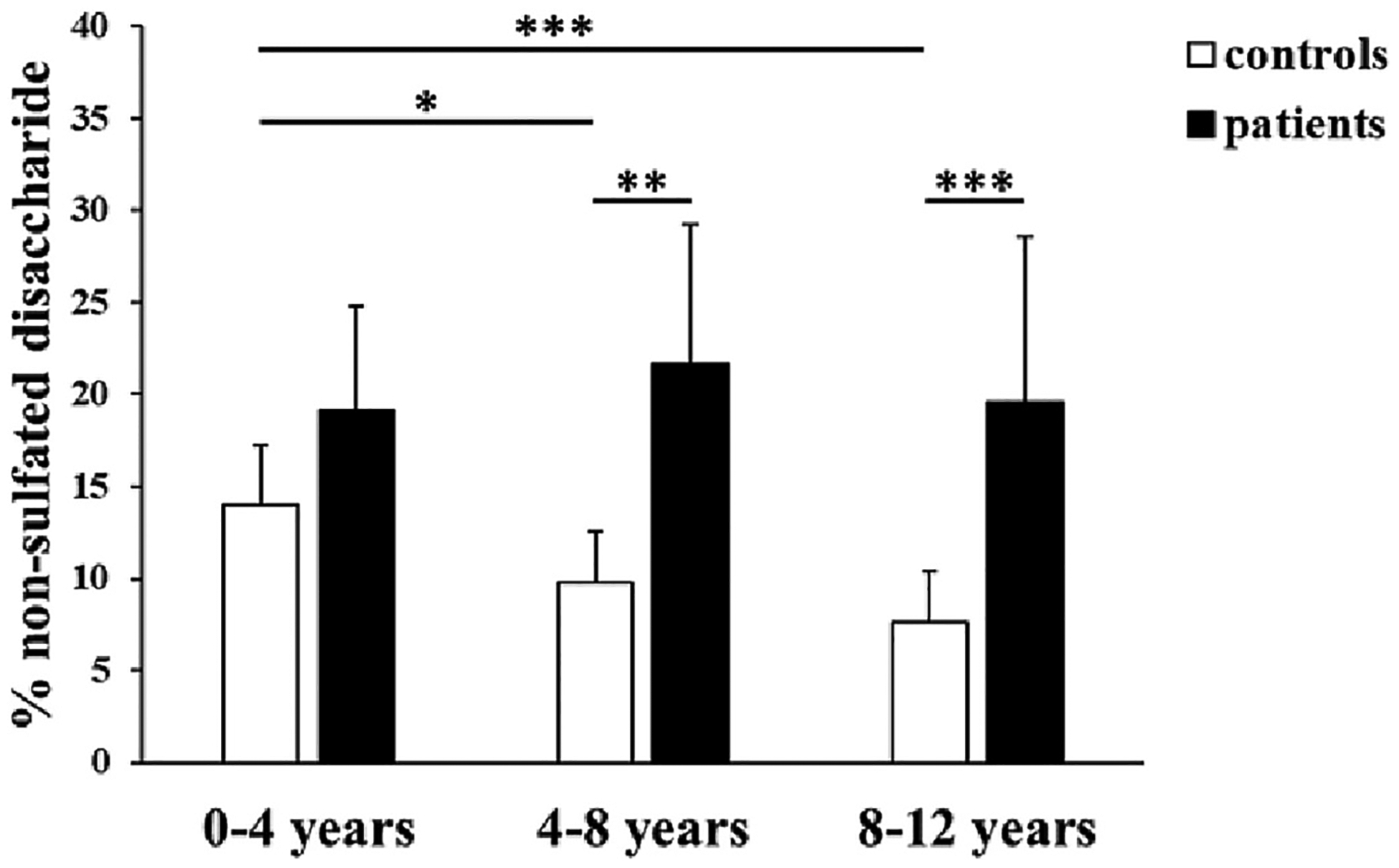
Urinary GAG sulfation analysis in controls and affected individuals grouped by age: 0–4, 4–8 and 8–12 years old. In controls, a decrease in the percentage of non-sulfated disaccharide was observed. The difference was significant between the 0–4 years old group and the other two groups. In the DTD study population, the percentage of non-sulfated disaccharide was higher than controls across the three age groups, but the difference was significant in the 4–8 and 8–12 years old groups. Data are reported as mean ± SD; 0–4 years old controls (*n* = 8) and DTD group (*n* = 6), 4–8 years old controls (n = 6) and DTD group (*n* = 5) and 8–12 years old controls (*n* = 13) and DTD group (n = 5). *P < 0.05; **P < 0.01; ***P < 0.001.

Since a moderate correlation between urinary GAG sulfation and age was identified, affected and unaffected individuals were grouped by age (0–4 years, 4–8 years and 8–12 years) to minimize the age effect during comparison. Among controls, the percentages of non-sulfated disaccharide were 14.0 % ± 3.3 % (*n* = 8; 0–4 years), 9.8 % ± 2.8 % (*n* = 6; 4–8 years) and 7.6 % ± 2.7 % (*n* = 13; 8–12 years). Within the control groups, a significant decrease in the relative amount of non-sulfated disaccharide was observed between the 0–4 years old group and the other two groups (*P* = 0.0267 and *P* = 0.00013, Fig. 2).

**Fig. 3.**
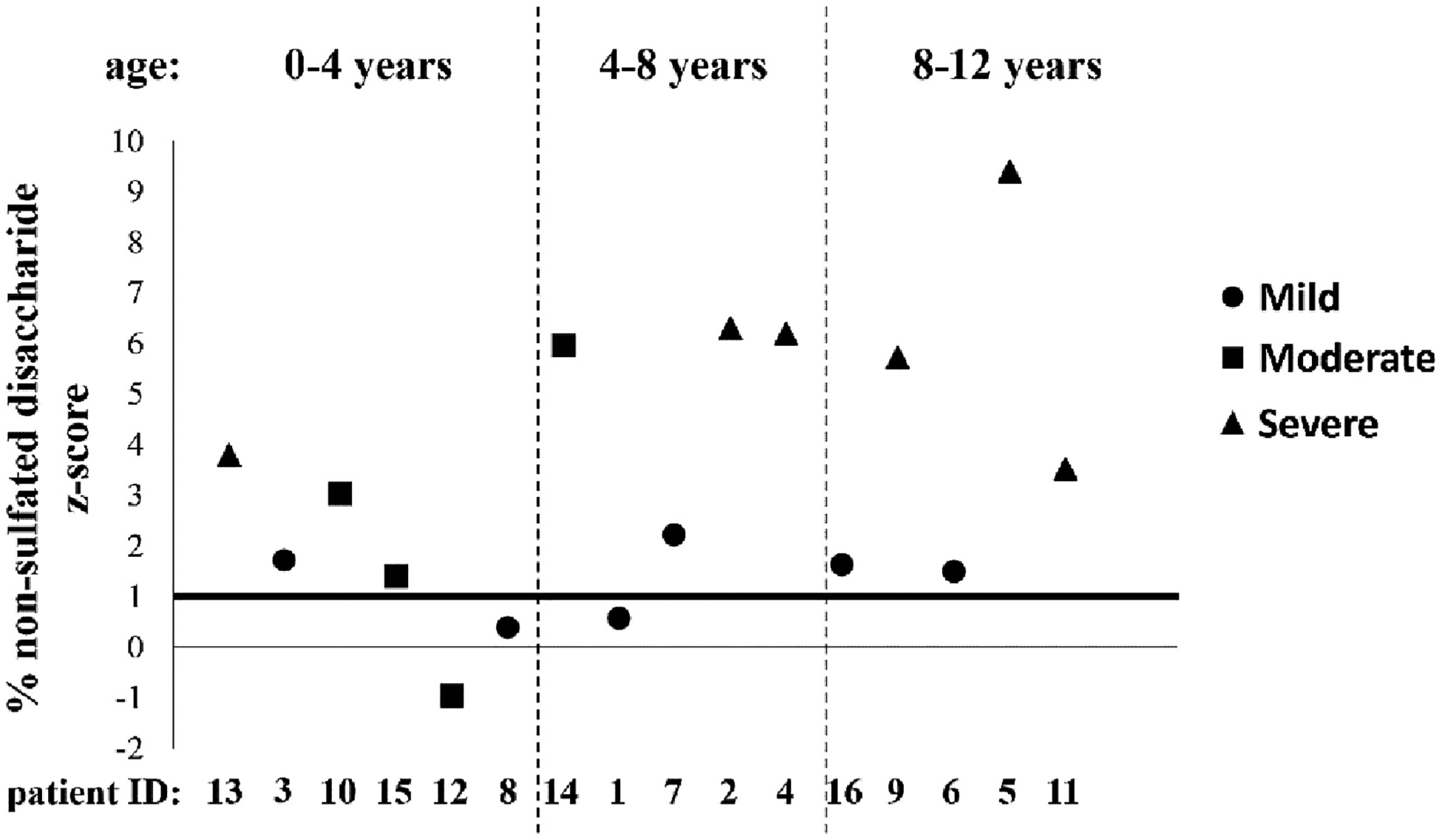
Z-score of the percentage of non-sulfated disaccharide in urine of affected individuals in relation with the clinical phenotype. For each individual the z-score was calculated considering mean and standard deviation of the corresponding age control group and the disease phenotype was classified in mild, moderate and severe based on the clinical features reported in Table 1. The highest z-scores were observed among 4–12 years old DTD individuals. Interestingly, DTD individuals with high z-scores presented a severe phenotype, while a mild phenotype was observed in DTD individuals with low z-scores, suggesting a possible correlation between urinary GAG sulfation and disease phenotype.

In the DTD cohort, the mean percentages of non-sulfated disaccha-ride were 19.1 % ± 5.7 % (n = 6; 0–4 years), 21.7 % ± 7.5 % (*n* = 5; 4–8 years) and 19.5 % ± 9.0 % (n = 5; 8–12 years). Among the three age groups, the relative amount of the non-sulfated disaccharide was significantly higher in the 4–8 years and 8–12 years old groups compared with age-matched controls (*P* = 0.006 and *P* = 0.0004), indicating GAG undersulfation. The percentage of non-sulfated disaccharides did not significantly decrease among the age groups in the DTD cohort, contrary to what observed in control samples (Fig. 2). Among the 16 individuals with DTD, 13 showed a z-score greater than +1 when compared to each age-matched control group (Fig. 3). Interestingly, the highest z-scores were observed in affected individuals aged 4–12 years, when in controls, the relative amount of non-sulfated disaccharide decreases.

Even if it is difficult to trace a correlation between the urinary GAG sulfation and the disease phenotype due to the age and gender heterogeneity within the patient cohort, based on the clinical observations summarized in Table 1 we divided DTD individuals in three groups of disease severity: mild, moderate and severe. Interestingly, DTD individuals classified as severe showed high z-scores of the percentage of non-sulfated disaccharide, while low z-scores corresponded to DTD individuals with mild or moderate phenotypes (Fig. 3). Moreover, the highest prevalence of severe phenotype was present among groups with children aged 4–12 years old, when the percentage of non-sulfated disaccharide was significantly increased in DTD individuals compared with controls. These observations suggest that urinary GAG sulfation could correlate with the disease severity.

Furthermore, urinary GAG sulfation analysis in the available family members of affected individuals showed that those with DTD had a higher percentage of non-sulfated disaccharide when compared with unaffected parents and siblings (Supplementary Fig. 1).

Overall, a marked urinary GAG undersulfation was demonstrated in individuals with DTD.

### 3.3. CXM analysis

**Fig. 4.**
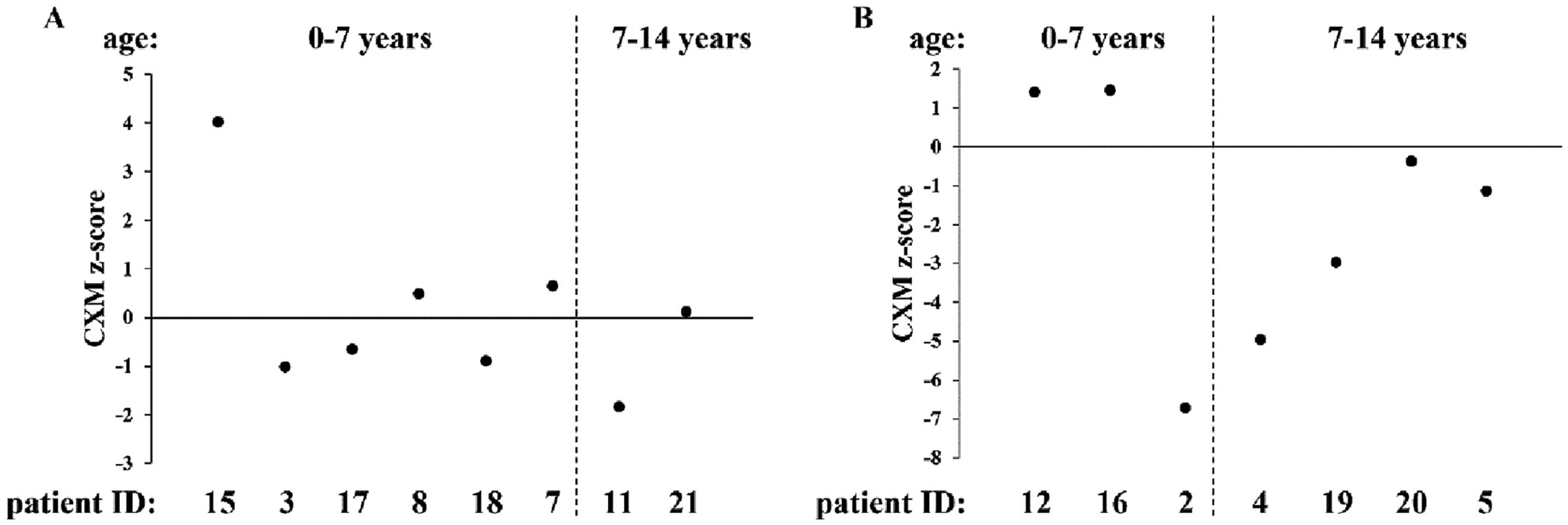
Z-scores of collagen X marker (CXM) in individuals with DTD grouped by sex, (A) males and (B) females. Individual z-scores were compared with general population reference data.

For CXM analysis, DBS samples from 15 DTD individuals with ages ranging from 0.5 to 14 years old were collected. The CXM z-score of each individual was calculated using age- and sex-matched reference data [22]. Results were categorized by sex (Fig. 4A, males and Fig. 4B, females) and grouped by age, 0–7 years and 7–14 years old, to include the pubertal growth spurt of both genders in the latter age group. The majority of data points were at or below the mean when compared with the general population; in particular, the most negative z-scores were observed in females with DTD. While the sex matched reference data and control curves were generated using serum and plasma samples, Coghlan et al. determined that serum, plasma, and DBS samples taken at the same time (matched samples) yield statistically identical CXM results when assayed [27]. Therefore, CXM data from DBS samples can be compared directly to control data curves for analysis. Interestingly, a difference in the relationship between growth velocity and blood CXM concentration was observed in DTD individuals compared with the reference population (Supplementary Fig. 2).

## 4. Discussion

In the last decades the enormous implementation in next generation sequencing techniques allowed the identification of a plethora of new genes causing various skeletal dysplasias fostering the elucidation of their pathomolecular mechanisms. Functional studies involving *in vitro* and *in vivo* models have also allowed for the development of potential targeted treatments of these conditions. The purpose of non-invasive biomarker identification is crucial, since drug development, subsequent clinical trials and the evaluation of treatment efficacy are costly and time consuming.

Clinical urinalysis is one of the most common non-invasive medical diagnostic methods. Aside from diabetes mellitus, other conditions including MPS, a group of lysosomal storage disorders, can be diagnosed and/or monitored through analysis of different types of carbohydrates. Various types of MPS can be identified based on the GAG content and species [17]. MPS VI, also known as Maroteaux-Lamy syndrome, is caused by deficiencies in *N*-acetylgalactosamine-4-sulfatase resulting from pathogenic variants in the arylsulfatase B (*ARSB*) gene and results in the accumulation of 4-sulfated chondroitin and dermatan sulfates [29]. Enzyme replacement therapy both in a MPS VI mouse model and in affected individuals reduces the level of 4-sulfated-disaccharide in urine suggesting that chondroitin sulfate disaccharide analysis might be a useful biomarker for future treatment monitoring [18].

Diastrophic dysplasia belongs to the *SLC26A2* family of disorders characterized by pathogenic variants in a sulfate transporter of the cell membrane required for sulfate uptake. Intracellular sulfate is crucial for cartilage and endochondral bone formation; thus, an impairment of sulfate uptake causes the chondrodysplastic phenotype. Chondroitin sulfate PG undersulfation has been demonstrated in cartilage biopsies and chondrocyte cultures of affected individuals and in the dtd mouse, a validated animal model of this dysplasia [5,30]. As urinary GAGs result from the turnover of PGs in the ECM, urine GAG sulfation was decreased in 1- and 2-month-old dtd mice compared with wild-types [21]. In a further work aimed at evaluating the efficacy of *N*-acetylcysteine on the skeletal phenotype of dtd newborns, increased urinary GAG sulfation was demonstrated in dtd mice born from females treated with the drug compared with the placebo group [13]. Overall, these findings suggested that urinary GAG sulfation could be a useful biomarker to monitor future pharmacological treatments. Based on these observations, we measured chondroitin sulfate sulfation of urinary GAGs in 16 individuals with DTD between 0.2 and 12 years old and their age-matched controls. Within the control group, we first evaluated whether gender differences were present in urinary GAG sulfation as pubertal growth spurt occurs between 10 and 12 years in males and 9–11 years in females [31]. Results demonstrated that no gender differences were present, thus simplifying future analysis. In 13 of 16 affected individuals, urinary GAGs were undersulfated suggesting that this biomarker may be a useful tool in assessing the efficacy of future treatments targeted at increasing PG sulfation.

Collagen X is synthesized by hypertrophic chondrocytes in growing individuals during endochondral growth plate ossification. CXM is a byproduct of collagen X degradation, which is released in blood. It is a biomarker of height velocity in typically growing individuals with its concentration in serum correlated to growth velocity [32]. CXM levels were significantly decreased in a cohort of individuals with achondroplasia younger than 18 years. The same analysis performed in those with hypochondroplasia demonstrated that CXM levels were not statistically different than controls, whereas individuals with thanatophoric dysplasia showed very low levels of CXM demonstrating a correlation between the degree of FGFR3 overactivity resulting in varying clinical phenotypes of FGFR3-related skeletal disorders and biomarker concentration [23]. CXM was measured in serum as an exploratory marker of growth plate activity directly related to the use of Vosoritide in children with achondroplasia. During the 24-months treatment period, median CXM concentrations were above baseline suggesting an increase in growth plate activity induced by Vosoritide [33]. CXM was evaluated in children with osteogenesis imperfecta (OI) type I and types III/IV. However, there was greater variability across the OI cohorts and the relationship with growth velocity was weaker; this may reflect heterogeneity in the manifestation of collagen I pathogenic variants within the growth plate [24].

In DTD, defects in the articular cartilage as well as in the growth plate were reported [34]. Moreover, alterations in growth plate PG sulfation as well as in chondrocyte proliferation and differentiation were demonstrated in the dtd mouse [35]. For this reason, we analyzed CXM level in DTD individuals between the age of 0.5 to 14 years old. In most individuals, the biomarker was below the age-matched control values likely reflecting abnormalities in the growth plate. Interestingly, patient females deviate more from the general population than males. Notably the same sex differences were reported in individuals with achondroplasia [23]. When CXM levels were plotted against height velocity in individuals with diastrophic dysplasia as compared with an unaffected cohort, there was an altered slope and y-intercept, suggesting different physeal dynamics. However, since CXM levels are highly dependent on age and growth velocity to establish a clearer pattern and a correlation to the DTD clinical severity a wider patient cohort might be necessary.

Although the CXM and urinary GAG sulfation analysis were performed in two different cohorts, these two biomarkers were significantly altered in children with DTD when compared with age-matched controls. In some individuals, both markers were analyzed, but it was difficult to trace a correlation among them which may reflect the different aspects of ECM metabolism. In fact, CXM is associated with alterations of the growth plate and of epiphyseal function, whereas urinary GAG sulfation correlates with chondroitin sulfate PG metabolism which mostly occurs in articular and growth plate cartilage.

In conclusion, both biomarkers may be useful as non-invasive approaches to assess the efficacy of treatments for DTD. In particular, CXM analysis may be suitable for longitudinal assessment of possible growth response within such targeted therapy. Alternatively, urinary GAG sulfation analysis appears to be a promising biomarker for therapeutic approaches aimed at increasing macromolecular sulfation by replenishing the intracellular sulfate pool.

## Supplementary Material

1

## Figures and Tables

**Table 1:** Clinical data of DTD affected individuals.

Patient ID	Analyzed biomarker	Origin	Sex	Disease severity	Nucleotide change	Predicted protein change	Age (years)	Height				Respiratory support		Cleft	Cervical kyphosis		Kyphoscoliosis			Cobb angle				Feeding support
								≤− 1SD	−1SD ÷ mean	mean ÷ +1SD	≥+1SD	CPAP	Tracheostomy/ventilator support		<60°	>60°	Present not requiring intervention	Requires bracing	Requires surgery	<10°	10–25°	25–40°	>40°	
1	U	AU	F	Mild	c.1994A > C c.835C > T	His665Pro Arg279Trp	5.0				√				√		√			√				
2	U/B	AU	F	Severe	c.835C > T c.1724delA	Arg279Trp Lys575S*fs*Ter10	6.7		√			In the past		√	√				√				√	
3	U/B	AU	M	Mild	c.1957 T > A c.2058C > A	Cys653Ser Cys686Ter	0.5				√				√									
4	U/B	AU	F	Severe	c.700-1G > A c.835C > T	Splice variant Arg279Trp	7.5		√			In the past		√	√				√				√	
5	U/B	AU	F	Severe	c.700-1G > A c.835C > T	Splice variant Arg279Trp	10.7	√				In the past		√	√				√				√	
6	U	CH	F	Mild	c.835C > T c.835C > T	Arg279Trp Arg279Trp	9.5				√													
7	U/B	USA	M	Mild	c.835C > T c.1994A > C	Arg279Trp His665Pro	6.1				√				√			√				√		
8	U/B	USA	M	Mild	c.835C > T c.1994A > C	Arg279Trp His665Pro	3.9				√				√									
9	U	USA	F	Severe	c.532C > T c.835C > T	Arg178Ter Arg279Trp	8.3	√					√	√		√			√				√	√
10	U	USA	M	Moderate	c.1535C > A c.699 + 2T > C	Thr512Lys Splice variant (“Finnish mutation”)	2.8			√					√				√				√	√
11	U/B	USA	M	Severe	c.699 + 3A > C c.699 + 3A > C	Splice variant	11.3	√					√	√		√			√				√	√
12	U/B	USA	F	Moderate	c.700-1G > C c.1957 T > A	Splice variant Cys653Ser	3.4	√							√				√				√	√
13	U	USA	M	Severe	c.835C > T c.1650delG	Arg279Trp Ser551Val*fs*Ter34	0.2		√							√								
14	U	USA	M	Moderate	c.532C > T c.835C > T	Arg178Ter Arg279Trp	5.0		√						√							√		
15	U/B	USA	M	Moderate	c.835C > T c.1654delT	Arg279Trp Ser552His*fs*Ter33	3.2		√					Submucosal cleft/bifid uvula	√			√			√			
16	U/B	USA	F	Mild	c.835C > T	Arg279Trp	8.1				√							√				√		
17	B	USA	M	Severe	c.2032_2033delinsCT c.835C > T c.1982delC	Gly678Leu Arg279Trp Thr661Lys*fs*Ter7	1.0	√						√		√		√					√	
18	B	USA	M	Severe	Pathogenic variant data not available		2.0		√				√	√					√				√	√
19	B	USA	F	Mild	Pathogenic variant data not available		8.6			√		√			Not noted in 2015 (no prior records available)		√				√			
20	B	USA	F	Moderate	Pathogenic variant data not available		9.2	√							Not noted in 2009 (no prior records available)			√				√		
21	B	USA	M	Mild	Pathogenic variant data not available		13.7			√					√		√				√			

U, urinary GAG sulfation analysis; B, blood CXM assay; SD, standard deviation; CPAP, continuous positive airway pressure.

## Data Availability

Data will be made available on request.

## Abbreviations:

ACG1B: achondrogenesis type 1B
AMAC: 2-aminoacridone
AO2: atelosteogenesis type 2
CXM: collagen X marker
DTD: diastrophic dysplasia
DTDST: diastrophic dysplasia sulfate transporter
ΔDi-0S: 3-O-β-(D-gluc-4-ene-uronosyl)-N-acetylgalactosamine
ΔDi-4S: derivative of ΔDi-0S with a sulfate at the 4 position of the hexosamine moiety
ΔDi-6S: derivative of ΔDi-0S with a sulfate at the 6 position of the hexosamine moiety
ECM: extracellular matrix
EMA: European Medicines Agency
FDA: Food and Drug Administration
FGFR3: fibroblast growth factor receptor 3
GAG: glycosaminoglycan
HPLC: high-performance liquid chromatography
MPS: mucopolysaccharidoses
NAC: *N*-acetyl-L-cysteine
OI: osteogenesis imperfecta
PG: proteoglycan
PLGA-PEG: polyethylene glycol-poly-lactide-co-glycolide block copolymer
rMED: recessive multiple epiphyseal dysplasia
SD: standard deviation
SLC26: solute carrier family 26
