# Supplementary material for: Identification of potential non-invasive biomarkers in diastrophic dysplasia
Source: Bone. Author manuscript; Available in PMC 2024 Dec 13. (PMC11638977; doi:10.1016/j.bone.2023.116838)
Supplement: 1 [file NIHMS2040248-supplement-1.docx]

**SUPPLEMENTARY**

**Identification of potential non-invasive biomarkers in diastrophic dysplasia**

Chiara Paganini, Ricki S. Carroll, Chiara Gramegna Tota, Andrea J. Schelhaas, Alessandra Leone, Angela L. Duker, David A. O’Connell, Ryan F. Coghlan, Brian Johnstone, Carlos R. Ferreira, Sabrina Peressini, Riccardo Albertini, Antonella Forlino, Luisa Bonafé, Ana Belinda Campos-Xavier, Andrea Superti-Furga, Andreas Zankl, Antonio Rossi, Michael B. Bober

**Supplementary Fig. 1**

**

**

**Urinary GAG sulfation analysis in the families of DTD individuals.** In all ten tested families, DTD individuals (black bars) showed a higher percentage of non-sulfated disaccharide when compared to unaffected parents and siblings (white bars). This result confirmed the marked GAG undersulfation in DTD individuals suggesting that urinary GAG sulfation analysis is a reliable non-invasive biomarker for DTD.

**Supplementary Fig. 2**


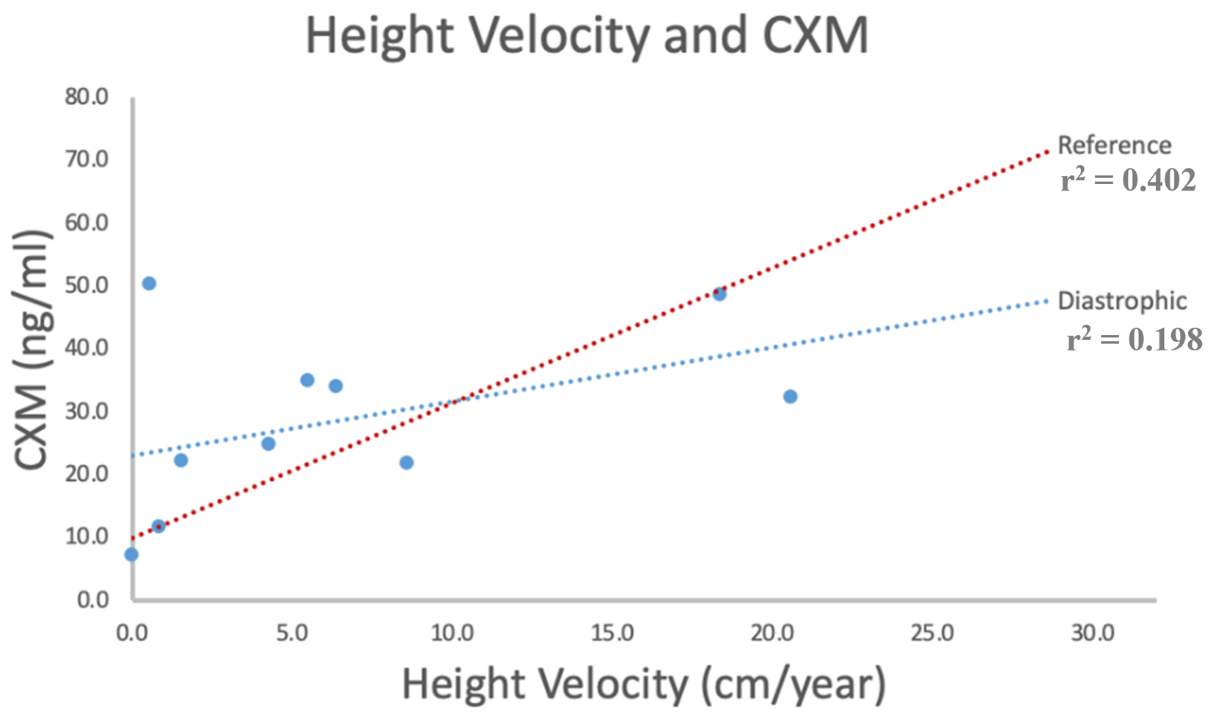


**CXM plotted as a function of growth velocity.** Data was plotted for all individuals with a CXM measurement and documented height velocity. A linear least squares regression line was calculated and compared to the normative data from Coghlan et al. (J. Clin. Endocrinol. Metab. 2021; 106(1)). The y-intercept and slope of the regression lines differ, suggesting there is in fact a difference in the relationship between growth velocities and CXM circulating levels compared to typically growing individuals.
